# Phase Engineering for Highly Efficient Quasi-Two-Dimensional All-Inorganic Perovskite Light-Emitting Diodes via Adjusting the Ratio of Cs Cation

**DOI:** 10.1186/s11671-019-3076-x

**Published:** 2019-07-27

**Authors:** Xiaoqiang Xu, Zijun Wang, Junsheng Yu, Lu Li, Xingwu Yan

**Affiliations:** 1 State Key Laboratory of Electronic Thin Films and Integrated Devices, School of Optoelectronic Science and Engineering, University of Electronic Science and Technology of China (UESTC), Chengdu, 610054 People’s Republic of China; 2 Research Institute for New Materials Technology, Chongqing University of Arts and Sciences, Chongqing, 402160 People’s Republic of China

**Keywords:** Perovskite light-emitting diodes, Quasi-2D, Phase engineering, Phenylpropylammonium

## Abstract

Quasi-two-dimensional (2D) perovskites have received intensive attention as a new class of luminescent materials owing to large exciton binding energy and high photoluminescence efficiency. However, there usually contains a mixture of phases in these materials, and excessive low-dimensional phase perovskite is harmful for luminescence efficiency owing to the strong exciton-phonon quenching at the room temperature. Herein, a simple and effective method is proposed to suppress the growth of low-dimensional phase components in quasi-2D perovskite film via carefully adjusting the molar ratio of cesium bromide (CsBr) and phenylpropylammonium bromide (PPABr). The device based on this optimized film has achieved a peak brightness of 2921 cd m^−2^ and peak current efficiency of 1.38 cd A^−1^, far away higher than that of the pristine CsPbBr_3_ device. This research proves a new way for modulating the phase composition in quasi-2D perovskites to fabricate highly efficient perovskite light-emitting diodes (PeLEDs).

## Introduction

Perovskite materials have aroused intensive research interests in thin-film light-emitting diodes owing to their exceptional optoelectronic properties, such as easily tunable emission wavelength [1, 2], high ambipolar charge mobility, facile solution processability, and low material cost [3–7]. But relatively low exciton binding energy and poor film-forming ability have resulted in inferior emission properties [8]. To circumvent these problems, many strategies have been adopted to boost the luminous efficiency in PeLEDs, such as composition modulating [9–12], interface engineering [13–16], nanocrystal pinning [17], solvent engineering [18–22], and polymer doping [23–25]. The external quantum efficiency (EQE) of the latest PeLEDs has been approaching 20%, nearly comparable to that of the current OLED [26, 27], which shows its great potentials for lighting and display applications.

Recently, quasi-2D perovskites, generally known as L_2_(CsPbX_3_)_*n* − 1_PbX_4_, have become the research hot materials in PeLEDs owing to high photoluminescence quantum efficiency (PLQY) and significantly improved stability compared to three-dimensional (3D) perovskite [28–36]. In these materials, the introduced alkyl or phenyl ammonium cations cannot fill into the interspace of [PbX_6_]^4−^ octahedral because of large ionic radius, resulting in the formation of layered perovskite film with self-assembly multiple-quantum-wells structure via spin-coating. In the quasi-2D perovskite structure, excitons are limited in inorganic layers to recombination due to big difference of permittivity between the incorporated ammonium barrier layers (L) and inorganic [PbX_6_]^4−^ octahedral layer, resulting in enlarged exciton binding energy [28]. Compared to 3D counterparts, quasi-2D perovskite films possess higher PLQY, smoother film morphology, lower defect-state density, and better environmental stability, which are beneficial for light-emitting applications [29]. For example, phenylethylammonium (PEA) cations were firstly used in green emission (PEA_2_MA_*n* − 1_Pb_*n*_Br_3*n* + 1_) with the maximum EQE of 8.8% and brightness of 2935 cd m^−2^ [28]. *n*-Butylammonium (BA) were introduced into MAPbBr_3_ perovskite precursor by Xiao et al. to obtain green PeLEDs with the EQE of 9.3% and a maximum brightness of 2900 cd m^−2^ [29]. Yang et al. reported the highly efficient green PeLEDs (PEA_2_FA_*n* − 1_Pb_*n*_Br_3*n* + 1_) with the EQE of 14.36% and peak luminance of 8779 cd m^−2^ based on perovskite films with *n* = 3 composition [34]. Recently, sky-blue PeLEDs with the peak brightness of 2480 cd m^−2^ were demonstrated based on *n* = 3 composition with double organic ammonium cations PEA and isopropyl ammonium (IPA) doping [35]. It has been demonstrated that the device based on quasi-2D perovskite with *n* = 3 composition can achieve high efficiency, but there exists a mixed phase in stoichiometric *n* = 3 composition perovskite [28, 34–37], which usually causes low emission efficiency. How to improve the phase purity in the quasi-2D perovskite remains a challenge.

In this work, by incorporating extra Cs cation into *n* = 3 composition perovskite precursor, efficient quasi-2D PeLEDs based on phenylpropylammonium bromide (PPABr) and CsPbBr_3_ were fabricated. Compared to 3D CsPbBr_3_ perovskite film, quasi-2D perovskite films exhibit full coverage, smaller grain size, and lower roughness. Moreover, the introduction of extra Cs cations in the precursor not only suppresses the formation of low-dimensional phase (little *n*-value-phase) with poor luminous efficiency, but also passivates the defect states in resulting quasi-2D perovskite film. Hence, the prepared perovskite films exhibit remarkable PL properties. By employing the resulting perovskite films as the emitting layer, quasi-2D PeLEDs with a peak brightness of 2921 cd m^−2^ and current efficiency of 1.38 cd A^−1^ were achieved, nearly threefold of that of the device based on *n* = 3 composition perovskite film.

## Methods

Lead bromide (PbBr_2_; Alfa Aesar, 99.999%); dimethyl sulfoxide (DMSO; 99.5% anhydrous, J&K Chemicals); poly(3,4-ethylenedioxythiophene):polystyrenesulfonate (PEDOT:PSS; Heraeus, VP AI4083); 1,3,5-tris(2-N-phenylbenzimidazolyl) benzene (TPBi; > 99.9%); cesium bromide (CsBr; 99.9%); and phenylpropylammonium bromide (PPABr; > 99.5%) were purchased from Xi’an Polymer Light Technology Corp. All materials were used as received without further purification. The perovskite precursor solutions were prepared by mixing PPABr, CsBr, and PbBr_2_ in DMSO and stirred at 60 °C overnight with different molar ratios of 2:2:3, 2:3:3, 2:3.5:3, and 2:4:3, respectively. The concentration of PbBr_2_ of every sample was kept constant in 0.15 M.

The ITO/glass substrates were ultrasonically cleaned in detergent, deionized water, acetone, and isopropanol in sequence for 20 min, respectively. After drying at 80 °C for 40 min, the substrates were treated in a UV-Ozone oven for 20 min prior to device fabrication. PEDOT: PSS (filtered by 0.45-μm PTFE syringe filter before deposition) was spin-coated onto cleaned substrates at 2900 rpm for 60 s and then baked at 150 °C for 20 min in the atmosphere. After that, all substrates were transferred into a nitrogen-filled glovebox. The as-obtained perovskite precursors were spin-coated onto substrates at 3000 rpm for 90 s and annealed at 90 °C for 15 min. The thickness of perovskite is about 70 nm. Next, TPBi (40 nm), LiF (1 nm), and Al (100 nm) were successively thermally deposited to complete the device in vacuum evaporation chamber under the basic pressure of 4 × 10^−4^ Pa. The active area of every PeLED is 0.11 cm^2^.

The current density-luminance-voltage (*J*-*L*-*V*) characteristic curves were monitored via two programed Keithley 2400 measurement unit coupled to a calibrated silicon photodiode. Electroluminescence (EL) spectra were recorded with a Photo Research PR670 spectrometer. PeLED characterizations were conducted in a nitrogen-filled glovebox without encapsulation. The morphology of perovskite films was investigated employing a field-emission scanning electron microscope (FESEM; ZEISS GeminiSEM 300) and atomic force microscope (AFM; Agilent AFM 5500). Structure characterization of perovskite films was conducted using an X-ray diffraction (XRD; X’Pert PRO, PANalytical). Absorption spectra of perovskite films were measured with an Agilent Cary 5000 UV-Visible spectroscopy. The steady-state PL spectra and time-resolved PL (TRPL) decay curves were determined by employing a HITACHI F7000 and an Edinburgh FLS980 fluorescence spectrophotometer, respectively.

## Results and Discussion

### Perovskite Film Characterizations

**Fig. 1:**
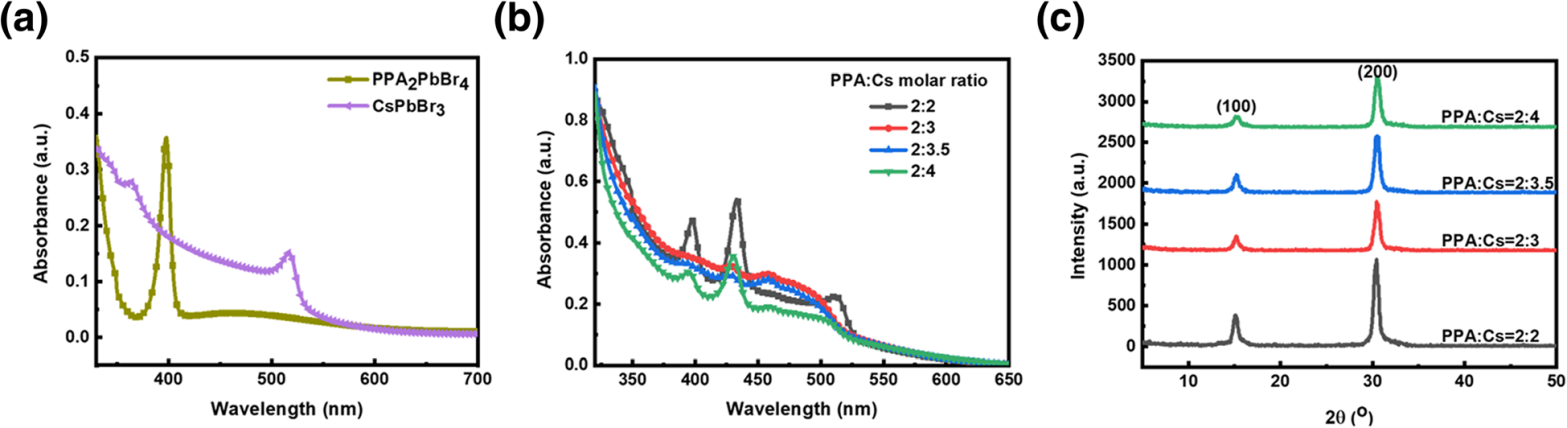
Absorption spectra in thin films of **a** the 3D CsPbBr_3_ and 2D PPA_2_PbBr_4_ perovskite, and **b** quasi-2D compounds with different molar ratios of PPA:Cs cations of 2:2, 2:3, 2:3.5, and 2:4. **c** XRD patterns of quasi-2D perovskite films with molar ratio of PPA:Cs cations of 2:2, 2:3, 2:3.5, and 2:4

The absorption spectra of perovskite films with different compositions are shown in Fig. 1a and b. From Fig. 1a, we can see CsPbBr_3_ film shows an absorption peak near 517 nm and PPA2PbBr4 film shows typical absorption peak at 400 nm, which corresponds to the *n* = 1 and *n* = *∞* phase perovskite, respectively, indicating that 2D perovskite has strong quantum confinement effects [28]. For perovskite films with different content of Cs cations, they all exhibit multiple absorption peaks, indicating that there indeed are mixed phase compositions in the four perovskite films [8, 34]. For *n* = 3 composition perovskite film (2:2), the exciton absorption peak corresponding to low-*n*-value phase perovskite was high, which means there exit large low-*n*-value phase in the perovskite film. However, when increasing the relative content of Cs ratio in precursor solutions (2:3 and 2:3.5), the absorption peaks belonging to middle *n*-phase perovskite began to appear, which turn out that lots of low-*n*-value phase perovskites have been transformed to large-*n*-value phase. To investigate the influence of extra Cs cations on perovskite crystalline properties, the X-ray diffraction (XRD) measurements were adopted. All films exhibited only two prominent diffraction peaks at 15.15° and 30.45°, respectively, which can be assigned to the (100) and (200) crystalline planes of orthorhombic phase CsPbBr_3_, indicating the preferential growth of perovskite crystallites, which is consistent with previous reports [30].

**Fig. 2:**
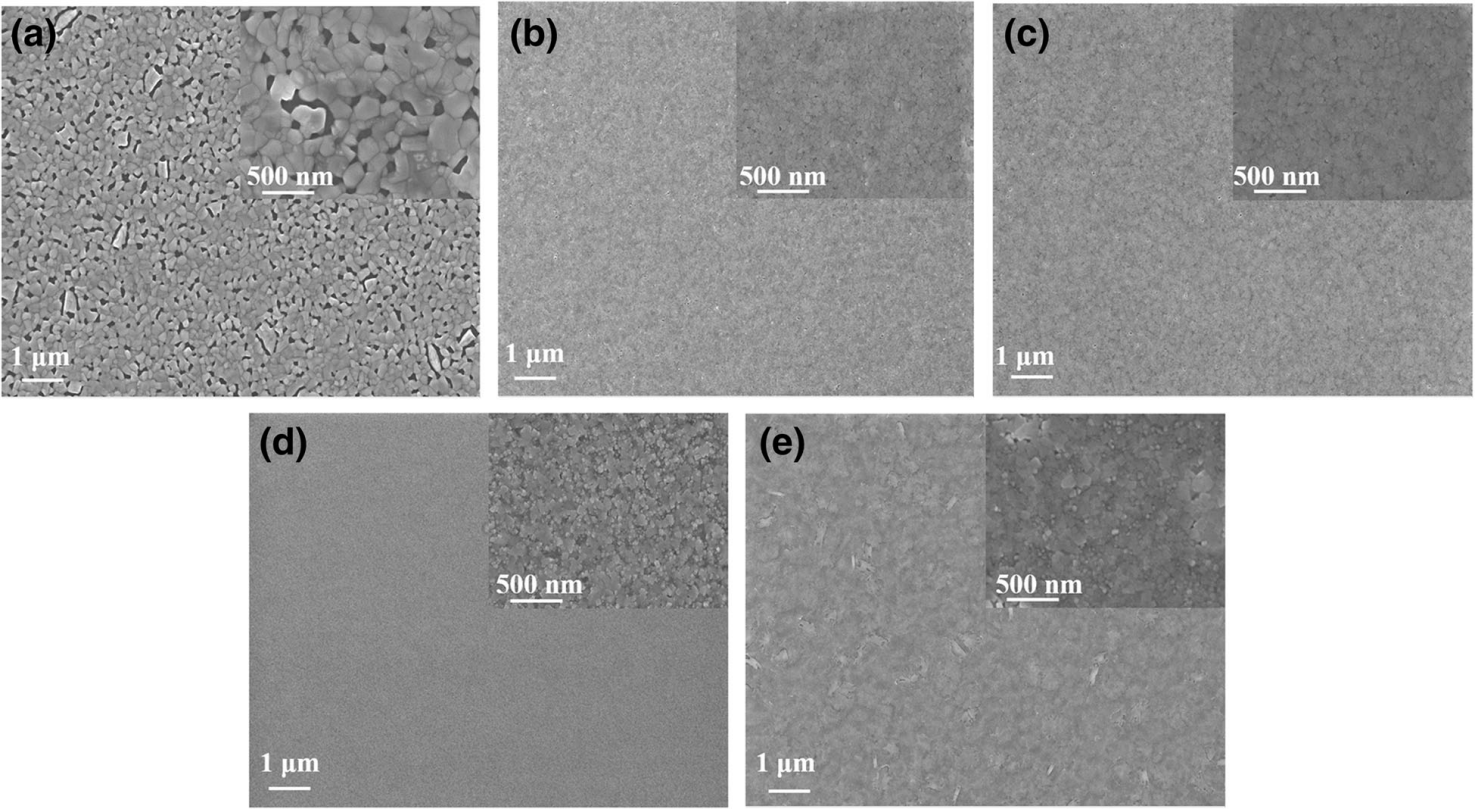
SEM images of perovskite films with **a** 3D perovskite and quasi-2D perovskite based on PPA:Cs cations of **b** 2:2, **c** 2:3, **d** 2:3.5, and **e** 2:4; the insets show the magnified picture of corresponding SEM. **b** AFM topographies of corresponding perovskite films

**Fig. 3:**
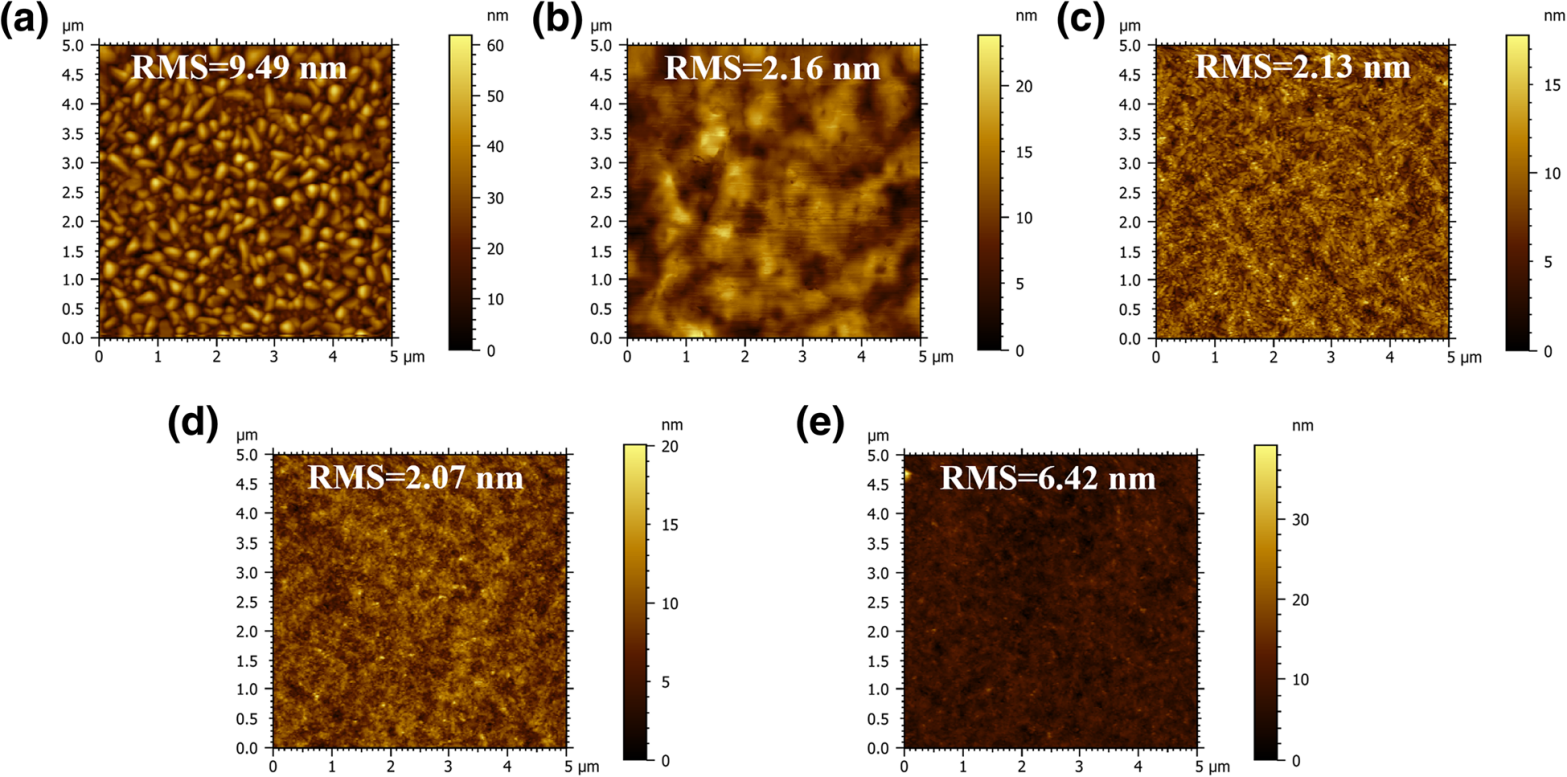
**b** AFM topographies of corresponding perovskite films with **a** 3D perovskite and quasi-2D perovskite based on PPA:Cs cations of **b** 2:2, **c** 2:3, **d** 2:3.5, and **e** 2:4

The morphological evolutions of perovskite thin films with different contents of Cs cations were recorded with SEM and AFM. From Figs. 2 and 3, we can see that pristine 3D CsPbBr_3_ show a poor surface morphology with many voids and big root-mean-square (RMS) roughness, which may cause electric shunt paths. In contrary, when PPABr is used, the film coverage is remarkably enhanced, and grain size is decreased sharply. The RMS of the pure 3D CsPbBr_3_ film is 9.49 nm, which is greatly decreased to 2.16 nm after the incorporation of PPABr (PPABr:CsBr = 2:2). When increasing the content of Cs cations to 2:3 and 2:3.5, the roughness remains at low levels. However, the surface became rough again when further increasing the Cs cation concentration to 2:4. These findings demonstrate that the incorporation of PPABr is indeed conducive to forming a compact and smooth thin film, and it could be found that incorporating Cs cations into precursor solution in an appropriate range has a little impact on perovskite thin film morphology.

**Fig. 4:**
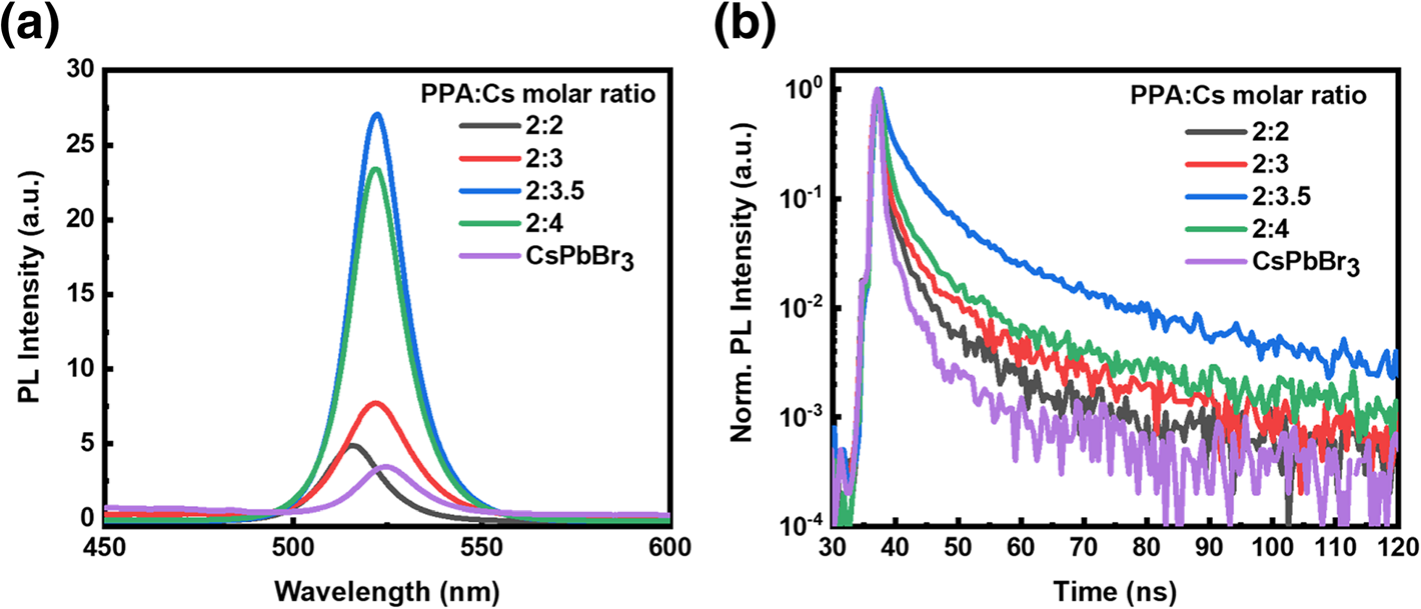
**a** PL spectra and **b** normalized TRPL decay curves of perovskite films with different molar ratios of PPA:Cs

Figure 4a exhibits the photoluminescence spectra of perovskite films with different molar ratios of PPA:Cs, which were measured to probe *n*-phase modulation in perovskite films. Obviously, photoluminescence emission peak gradually blueshifted from 524 nm for 3D CsPbBr_3_ thin film to 517 nm for perovskite thin films of 2:2, indicating an incremental quantum confinement effect. When increasing the relative content of Cs cations, the PL spectra show a slight redshift. Meanwhile, the perovskite film with the PPA:Cs molar ratio of 2:3.5 shows the highest PL intensity under the same excitation condition. To gain deep insight into the effect of Cs content in the precursor solution on exciton properties of perovskite films, time-resolved photoluminescence (TRPL) decay curves of perovskite films were measured and displayed in Fig. 4b, which can be well fitted by tri-exponential expression (1) [38]:

1 $$ I={A}_1{\mathrm{e}}^{-\frac{t}{\tau_1}}+{A}_2{e}^{-\frac{t}{\tau_2}}+{A}_3{e}^{-\frac{t}{\tau_3}} $$

in which *I* represents the normalized PL intensity; *A*_1_, *A*_2_, and *A*_3_ stand for the proportion of the components; and *τ*_1_, *τ*_2_, and *τ*_3_ represent respective exciton lifetime for different carrier kinetic process. The average lifetime (*τ*_avg_) is calculated in the following expression (2) [19]:

2 $$ {\tau}_{\mathrm{avg}}=\frac{A_1{\tau_1}^2+{A}_2{\tau_2}^2+{A}_3{\tau_3}^2}{A_1{\tau}_1+{A}_2{\tau}_2+{A}_3{\tau}_3} $$

where the *τ*_3_ component is ascribed to radiative recombination process in perovskite grains and *τ*_1_ and *τ*_2_ correspond to two types of trap-assisted recombination. Table 1 summarizes the fitted parameters of the three-exponential fitting result of TRPL decays. The average time for a pristine 3D CsPbBr_3_ sample is small (7.02 ns). But it is improved significantly by introducing PPA, which is attributed to greatly enlarged exciton binding energy [29]. And when increasing the Cs cation content in the precursor solution, the *τ*_avg_ of 2:3.5 shows the largest average lifetime of 32.11 ns, indicating that there is decreased defect-state density compared to perovskite films with other compositions, in combination with the similar surface morphology and absorption spectra. According to the above discussion, it can be concluded that appropriate Cs cations in perovskite precursor can impede the growth of the low-*n*-perovskite phase perovskite [37] and led to decreased trap density and prolonged carrier lifetime.

**Table 1:** Detailed fitted parameters of time-resolved photoluminescence decay curve

PPA:Cs	*A*_1_ (%)	*τ*_1_ (ns)	*A*_2_ (%)	*τ*_2_ (ns)	*A*_3_ (%)	*τ*_3_ (ns)	*τ*_avg_ (ns)
2:2	40.73	0.55	41.52	3.17	17.75	19.49	14.35
2:3	32.98	1.40	42.52	6.15	24.51	30.48	23.17
2:3.5	28.72	1.69	50.87	8.26	20.42	44.70	32.11
2:4	53.32	0.72	32.79	4.93	13.90	41.67	32.03
CsPbBr_3_	76.64	0.32	18.24	2.17	5.12	12.56	7.02

### LED Device Fabrication

**Fig. 5:**
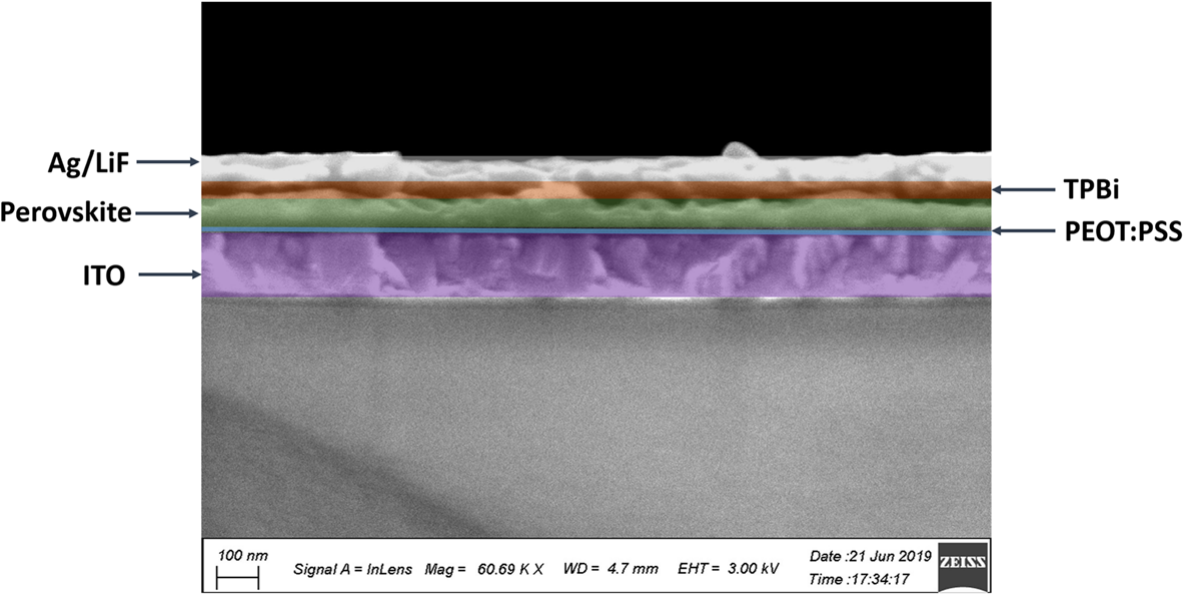
Cross-sectional field-emission SEM of a quasi-2D PeLED

**Fig. 6:**
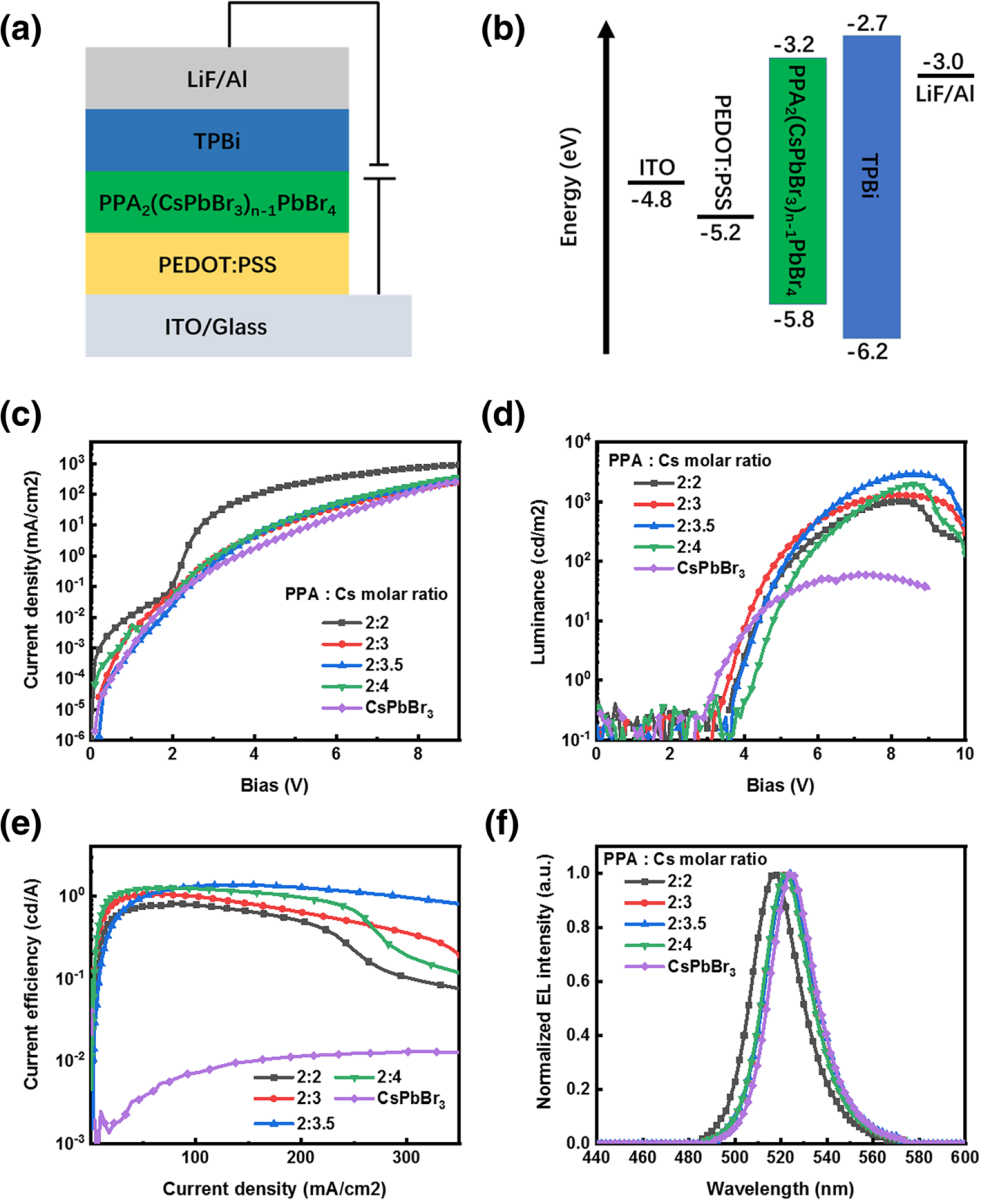
**a** Schematic diagram of the device structure and **b** corresponding energy level for the PeLEDs. **c** Current density versus voltage curves (J-V), **d** luminance versus voltage (L-V), and **e** current efficiency versus voltage (CE-V) characteristic curves of PeLEDs based on different perovskite films. **f** Normalized EL spectra

Employing abovementioned perovskite films as an emitting layer, perovskite LEDs (ITO/PEDOT: PSS/PPA_2_(CsPbBr_3_)_*n* − 1_PbBr_4_/TPBi/LiF/Al) were fabricated, as shown in Figs. 5 and 6a and b. Figure 6c–e displays current density, luminance, and current efficiency as a function of voltage (*J*-*V*, *L*-*V*, and CE-*V*) characteristic curves for the devices with different molar ratios of PPA:Cs cations. It can be clearly seen that the incorporation of PPABr leads to the obvious decrease in leakage current under low applied voltages, demonstrating remarkably reduced shunting paths in perovskite film, in good agreement with the morphology characterization results mentioned above. As shown in Fig. 6d and e, the device with PPA:Cs molar ratio of 2:2 shows the significantly improved peak brightness of 1026 cd m^−2^ compared with that of 60 cd m^−2^ for 3D CsPbBr_3_-based device, and the current efficiency improved from 0.01 to 0.80 cd A^−1^. With further improvement in the Cs cations in the perovskite precursor solutions, the maximum luminance and current density got further improvements, of which the device with the PPA:Cs molar ratio of 2:3.5 exhibits the peak luminance of 2921 cd m^−2^, which is nearly threefold improvement compared with that of the device with the PPA: Cs molar ratio of 2:2, and the current density increased to 1.38 cd A^−1^. The electroluminescence spectra (Fig. 6e) of PeLEDs with different compositions all show slightly redshifted emission peaks compared to the corresponding PL peaks, which is consistent with previous reports [37, 38]. Concrete PeLED characterization results are summarized in Table 2. Significantly improved device performance could be ascribed to improved morphology and reduced proportion of low-dimensional phase perovskite resulting from the extra Cs cations.

**Table 2:** Summary of detailed PeLED performance parameters

PPA:Cs	*V*_turn-on_ (V)	*L*_max_ (cd m^−2^)	CE_max_ (cd A^−1^)	EQE_max_ (%)	EL peak (nm)
2:2	3.8	1026	0.80	0.004	517.0
2:3	3.6	1289	1.05	0.006	522.4
2:3.5	3.8	2921	1.38	0.01	523.0
2:4	4.2	1944	1.25	0.007	522.2
CsPbBr_3_	3.3	60	0.01	–	524.6

## Conclusions

In summary, a facile and efficient strategy to achieve high-performance perovskite LEDs via phase engineering has been developed. It is found that the introduction of organic spacer (PPABr) could remarkably reduce the domain size and increase the surface coverage of perovskite film. By further incorporating moderate cesium bromide into quasi-2D perovskite, the proportion of low-dimensional phase component in quasi-2D perovskite was significantly diminished, leading to a remarkably enhanced photoluminescence intensity and prolonged exciton lifetime. Hence, best performing PeLED based on the optimum Cs cation content shows a peak brightness of 2921 cd m^−2^ and a current efficiency of 1.38 cd A^−1^, respectively. It is believed that this method may provide a guide for improving the emission efficiency of PeLEDs with quasi-2D perovskite film.

## Data Availability

All datasets are presented in the main paper or in the additional supporting files.

## Abbreviations

2D: Two-dimensional
3D: Three-dimensional
AFM: Atomic force microscope
CE-V: Current efficiency-voltage
CsBr: Cesium bromide
EQE: External quantum efficiency
FESEM: Field-emission scanning electron microscope
ITO: Indium tin oxide
J-V: Current density-voltage
L-V: Luminance-voltage
PbBr_2_: Lead bromide
PeLEDs: Perovskite light-emitting diodes
PLQY: Photoluminescence quantum efficiency
PPABr: Phenylpropylammonium bromide
TRPL: Time-resolved photoluminescence
XRD: X-ray diffraction
*τ*_avg_: Average lifetime
